# Supercritical SC-CO_2_ and Soxhlet *n*-Hexane Extract of Tunisian *Opuntia ficus indica* Seeds and Fatty Acids Analysis

**DOI:** 10.1155/2012/914693

**Published:** 2012-06-17

**Authors:** Nizar Yeddes, Jamila Kalthoum Chérif, Amel Jrad, Danielle Barth, Malika Trabelsi-Ayadi

**Affiliations:** ^1^Laboratoire d'Application de la Chimie aux Ressources et Substances Naturelles et à l'Environnement (LACReSNE), Faculté des Sciences de Bizerte, Zarzouna, Bizerte 7021, Tunisia; ^2^Institut préparatoire aux Études d'Ingénieurs de Tunis (IPEIT), Montfleury, Tunis 1008, Tunisia; ^3^Centre Internationale de Technologie de l'Environnement (CITET), Boulevards Leader Yasser Arafat, Tunis 1080, Tunisia; ^4^Laboratoire Réactions et Génie des Procédés, UPR CNRS 3339, Nancy-Université, 1 rue Grandville, BP 20451, Nancy, France

## Abstract

The fatty acids profiles of Tunisian *Opuntia ficus indica* seeds (spiny and thornless form) were investigated. Results of supercritical carbon dioxide (SC-CO_2_) and soxhlet *n*-hexane extract were compared. Quantitatively, the better yield was obtained through soxhlet *n*-hexane: 10.32% (spiny) and 8.91% (thornless) against 3.4% (spiny) and 1.94% (thornless) by SC-CO_2_ extract (*T* = 40°C, *P* = 180 bar, time = 135 mn, CO_2_ flow rate = 15 mL**·**s^−1^). Qualitatively, the main fatty acids components were the same for the two types of extraction. Linoleic acid was the major compound, SC-CO_2_: 57.60% (spiny), 59.98% (thornless), soxhlet *n*-hexane: 57.54% (spiny), 60.66% (thornless), followed by oleic acid, SC-CO_2_: 22.31% (spiny), 22.40% (thornless), soxhlet *n*-hexane: 25.28% (spiny), 20.58% (thornless) and palmitic acid, SC-CO_2_: 14.3% (spiny), 12.92% (thornless), soxhlet *n*-hexane: 11.33% (spiny), 13.08% (thornless). The SC-CO_2_ profiles fatty acids showed a richness with other minority compounds such as C_20:1_, C_20:2_, and C_22_.The seeds oil was highly unsaturated (US = 4.44–5.25), and the rising temperatures donot affect the selectivity of fatty acids extract by SC-CO2: US = 4.44 (*T* = 40°C) and 4.13 (*T* = 70°C).

## 1. Introduction

*O. ficus indica* is a species of cactus that belongs to the *Cactaceae* family, the order *Centrospermae,* and the genus *Opuntia.* This plant is associated with the semiarid zones of the world mainly in Africa, Mediterranean countries, southwestern United States, northern Mexico, and other areas [1]. In Tunisia, the prickly pear grows everywhere and mainly used for human consumption [2]. In chemistry, the main studies on the *Opuntia* fruits were the chemical analysis of pulp, skin, and seeds [1]. The proximate composition of *O. ficus indica* L. has been investigated [1, 3]. Seeds constitute about 10–15% of edible pulp and are usually discarded in the food industry as waste after pulp extraction. Edible oil can be obtained from prickly pear seeds in yield of 5.8–13.6% [1, 4–6]. The oil has a high degree of unsaturation in fatty acids (82%) with a linoleic acid content of 73.4% [5]. According to the investigation of Ramadan and Mörsel [7] using a chloroform/methanol as extraction solvent procedure of total lipids in seeds oil of cactus pear (*O. ficus indica *L.), linoleic acid was the dominating fatty acid (53.5%), followed by palmitic (20.1%) and oleic acids (18.3%). Two spices of prickly pear from Tunisia, *O. ficus indica *and *O. stricta,* were investigated for fatty acid composition seeds by Ennouri et al. [2] with an exceptional level of linoleic acid (up to 70%); the oils were extracted with hexane in a soxhlet extractor. Moreover, no data has been mentioned about the difference of fatty acids composition in the two spines (wild) and thornless (cultivated) forms of Tunisian *O. ficus indica* seeds. Mechanical processes and organic solvent were used for extracting industrial seed oil. The oil obtained by mechanical separation processes was of high quality, but, in most cases, the yield was lower. Hexane extraction achieved almost complete recovery of the oil, and the resulting oil contains traces of solvent; however, the solvent was dangerous and unacceptable as it was quite harmful to human health and the environment, which may restrict its use in food, cosmetic and pharmaceutical industries. Supercritical fluid extraction (SFE) with SC-CO_2_ represents an alternative method for the extraction of oils from natural products and has received considerable attention [8]. SFEs have been used as solvents for a wide varieties of applications such as essential oil extraction [9]. SFE use increases in the last years because of legal limitations of solvent residues, and solvents make this process more economical. The CO_2_ remained the most commonly used fluid for SFE applications thanks to its low critical parameters (Tc = 31.1°C; Pc = 74 bar), it has non-toxic, nonflammable properties, and it was available in high purity with low cost. The oils obtained by SC-CO_2_ extraction were of outstanding quality, and the yields were comparable with those obtained with organic solvent extraction method [10]. The CO_2_ can be used in many extractions and reactions and has been recognized as earth-compatible solvent [11]. Therefore, SFE may serve as a promising technology in food and pharmaceutical processing [12]. The objective of the present research was to illustrate and to compare between the SC-CO_2_ and the soxhlet *n*-hexane profiles fatty acids of two forms of Tunisian *O. ficus indica* seeds.

### 1.1. Practical Applications

Research on oil extracted from *O. ficus indica* seeds showed that this material was a potential source of food and feed. Moreover, these oils have several applications in pharmacology, medicinal, and cosmetic fields. The fact that extraction with SC-CO_2_ enables to have pure oil free of solvent makes it possible to better enhance these oils safety.

## 2. Materials and Methods

### 2.1. Prickly Pear Seed

Fresh and mature pears fruits of two forms of Tunisian *O. ficus indica* were collected in summer 2009. The spiny wild form was from the region of Al-Ala in the centre of Tunisia located at 35° 36′ N (North) latitude, 9° 34′ E (East) longitude, and 450 m (meter) altitude. The thornless cultivated form was from local pilot cultivar of Bou Argoub region in the north east of Tunisia located at 36° 32′ N latitude, 10° 33′ E longitude, and 62 m altitude. The fruits were washed with running water, air dried, and hand peeled. The seeds were recovered, after mixing the pulp and filtration through a 2 mm sieve. The seeds were washed thoroughly with bidistilled water, dried in an oven at 35°C for 48 h. The seeds were ground by a domestic coffee grinder (*SEB Prep line*) just before extraction. The moisture content of the seeds was determined by drying 50 g of seeds at 70°C to a constant weight in an oven for 72 h.

### 2.2. Standards and Reagents

Reference standards of caprylic acid (C_8__:__0_), capric acid (C_10__:__0_), lauric acid (C_12 : 0_), myristic acid (C_14__:__0_), palmitic acid (C_16__:__0_), palmitoleic acid (C_16__:__1_), heptadecanoic acid (C_17__:__0_), stearic acid (C_18__:__0_), oleic acid (C_18__:__1_), linoleic acid (C_18__:__2_), linolenic acid (C_18__:__3_), arachidic acid (C_20__:__0_), eicosenoic acid (C_20__:__1_), eicosadienoic acid (C_20__:__2_) and behenic acid (C_22__:__0_) that had a minimum purity of 99%, sodium methoxide, methanol (HPLC grade), hexane, and sulfuric acid (1N) were purchased from Sigma Aldrich (USA). The SC-CO_2_ extractions were carried out with a high purity carbon dioxide (99.95%) supplied by Messer (France).

### 2.3. Supercritical Fluid Extraction

The extractions were conducted in a cylindrical stainless steel extractor (300 mm × 23 mm, internal volume: 125 cm^3^) and a three cyclonic separators type. The ground seeds (50 g) of *O. ficus indica* (spiny: 92.49%—thornless: 93.64% dry matter) were loaded into steel cylinder, filled into the extractor vessel and extracted at temperatures of 35, 40, 45, 50, 60, and 70°C (spiny) and 40°C (thornless), 180 bar and the CO_2_ flow rate of 15 mL·s^−1^ for 135 min. The desired extraction temperature was achieved by heating the extraction vessel, and the temperature was monitored by a thermocouple and regulated by a controller. The extraction pressure was controlled by a back pressure regulator, and the flow rate of CO_2_ was controlled by manual adjustment of a needle-metering valve. The extracts were collected in one tube throughout the 135 min, and the yields were calculated (for each temperature). The final volume of extract was analyzed using GC-FID to determine the fatty acids profile as described below.

### 2.4. Soxhlet Method Extraction

Soxhlet *n*-hexane extraction was carried in triplicate for 30 g of ground seed of *O. ficus indica* by 250 mL of *n*-hexane (99%) for 8 h. A rotary evaporator was used for the efficient removal of solvent (*n*-hexane) from samples by evaporation at 68°C. The extracts were stored in a glass jars in the deep freeze (−20°C) pending for further analysis.

### 2.5. Preparation of Fatty Acid Methyl Esters (FAMEs)

The extract samples were methylated by sodium methoxide according to the method of Carreau and Dubacq [13]. The methylation of fatty acids occurred in the presence of 0.5 mL sodium methoxide in methanol 3% and 2 mL of hexane. A known amount of methyl ester heptadecanoic acid (C_17__:__0_) was used as internal standard to quantify the fatty acids. After stirring during 60 seconds and decantation for 2 min, the reaction was neutralized by the addition of 0.2 mL of H_2_SO_4_ (1N). Finally, 1.5 mL salt water (10% NaCl) was added to allow the separation of two phases. The organic higher phase containing dissolved methyl esters in hexane was retained. After a concentration under nitrogen current, the FAMEs were analyzed by GC-FID.

### 2.6. Fatty Acid Analysis

The methyl-esterified fatty acids were analyzed by GC-FID to determine fatty acid constituents. The GC analysis was performed by using a Hewlett-Packard (HP) 6890 series equipped with a flame ionization detector (FID) and a polar capillary column: HP Innowax (30 m length × 0.25 mm internal diameter and 0.25 *μ*m film thickness).The column temperature was initially at 120°C (held for 1 min) and then increased to 200°C at 15°C min^−1^ and finally increased to 250°C at 2°C min^−1^. The operational conditions were injector temperature 250°C; detector temperature 275°C; carrier gas was nitrogen at a flow of 1.7 mL min^−1^ with a split ratio of 40 : 1. The fatty acids were identified by comparing their chromatograms to those of pure standards analyzed under the same conditions. The HP ChemStation software allowed the end of each analysis to provide the surface of each peak and its percentage. Three injections were done for quantification of each fatty acid. 

### 2.7. Statistical Analysis

All experiments were the result of three runs that were averaged together. The standard deviations of the extractions yields and the fatty acid constituents were based on triplicate measurements. Each value was expressed as mean ± standard deviation (SD).

## 3. Results and Discussion

In this study, we intend to compare the efficiency of the SFE by SC-CO_2_ extract process with the solvent extraction by soxhlet *n*-hexane. Efficiency is based on the extraction yield (quantity) with its relationship to composition of fatty acids (quality).

### 3.1. SC-CO_2_ and Soxhlet *n*-Hexane Extract Yield

**Figure 1:**
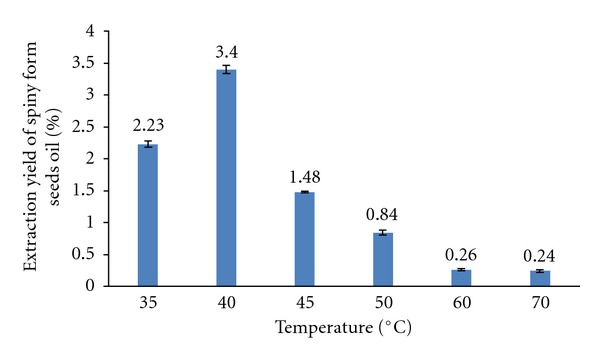
SC-CO_2_ extraction yield of seeds oil from Tunisian *O. ficus indica* spiny form versus temperature.

Various parameters potentially influenced the SC-CO_2_ extraction process, and the first step of SC-CO_2_ extract knowledge was to optimize the temperature at fixed conditions: Pressure = 180 bar, CO_2_ flow rate = 15 mL s^−1^, and time = 135 min, to obtain a better yield of extract. The experimental results of SC-CO_2_ extract yield of the spiny form were calculated and illustrated in Figure 1.

**Figure 2:**
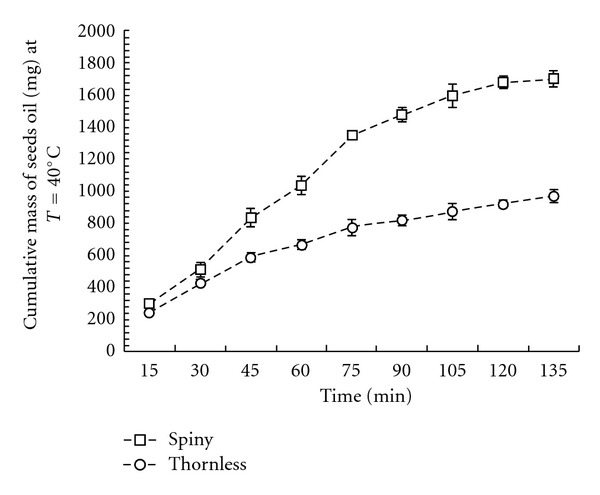
Cumulative SC-CO_2_ extraction yield oil of Tunisian *O. ficus indica* seeds at *T* = 40°C versus time.

The yields (*Y*) of oil defined on 100 g dry seeds of *O. ficus indica *basis and the total extraction yield were calculated as follow:

(1) $$\begin{array}{c} Y\%=\frac{\left[\left(m_{2}-m_{1}\right)\times 100\right]}{M}, \end{array}$$

where *m*_2_ was the mass of glass trap with extract, *m*_1_ was the mass of empty glass trap, and *M* was the initial mass of seeds in the extractor. The extract collected in the cold trap at atmospheric pressure consisted of a yellow solid mass and water. According to Figure 1, the extraction yield of seed oil depended most on extraction temperature variations. The extraction yields first increased from 1114.8 mg ± 34.5/50 g of ground seeds at *T* = 35°C, reached a maximum value at 40°C (1702.4 mg ± 40.1/50 g of ground seeds), and then decreased at higher temperatures 70°C (122.3 mg ± 6.6/50 g of ground seeds). Extraction with SC-CO_2_ at *T* = 40°C versus time of spiny and thornless forms of *O. ficus indica* seeds showed, respectively, an extraction percentage of 3.4% (1702.4 mg ± 40.1/50 g of ground seeds) and 1.94% (970.7 mg ± 49/50 g of ground seeds) (Figure 2). In the same condition of our extraction (*T* = 40°C and fixed parameters), the SC-CO_2_ extraction yields depended on the variability of plant material (seeds) of two *Opuntia *forms. At *T* = 40°C, the extraction yield was gradually increased with increasing time, for the two forms of *O. ficus indica*, the majority of the extract oil was recovered at an interval time of 15 to 75 min, and the extraction yields were 79.42% and 78.82%, respectively, for the spiny and the thornless form. The oils soxhlet *n-*hexane extract yield for the wild spiny form (10.32%) and cultivated form (8.91%) of Tunisian O*. ficus indica* were higher than the SC-CO_2_ extract. These values were similar to the results found by Ennouri et al. [2] (Table 1).

The performance of SC-CO_2_ extraction was relatively low compared to soxhlet *n*-hexane extract and other extraction methods that exist in the literature, the extraction solvent chloroform/methanol recovers 9.88% [7]. According to Snyder et al. [14], moisture content between 3% and 12% has very little effect on the extractability of oils from seeds with SC-CO_2_.

### 3.2. Fatty Acids Composition of Seed Oil

**Figure 3:**
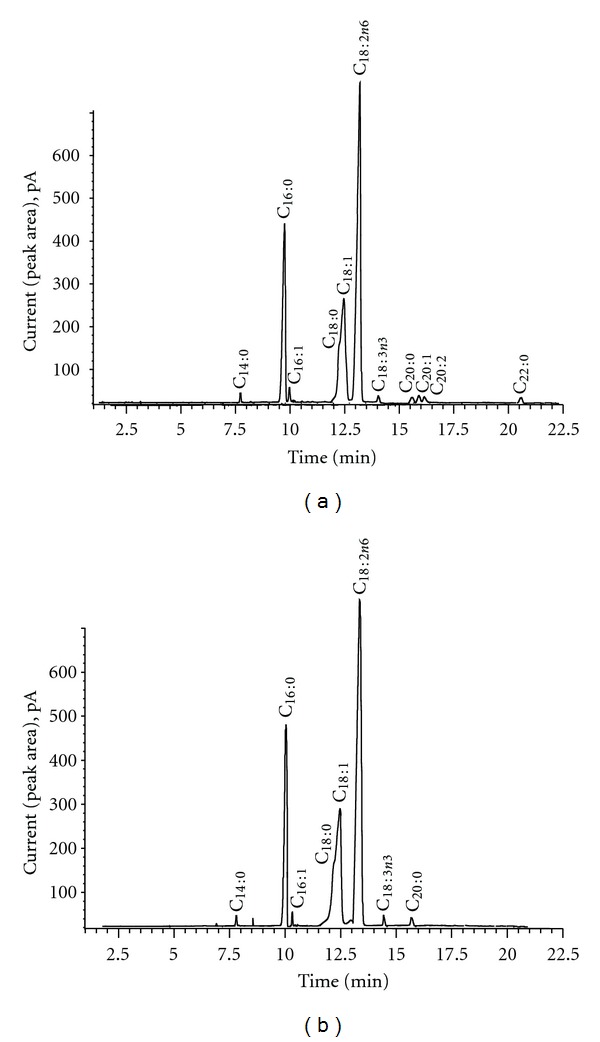
GC-FID chromatograms of fatty acids profile of Tunisian *O. ficus indica* seeds oil (spiny form), (a) from SC-CO_2_ extract (*P* = 180 bar, *T* = 40°C, CO_2_ flow rate = 15 mL·s^−1^ and time = 135 min) and (b) from soxhlet *n*-hexane extract.

In the literature, it was reported that *O. ficus indica* contain a good quality of edible oil [4, 5, 15, 16] such as fatty acids. The fatty acid profiles of two forms of *O. ficus indica* seeds were analyzed by GC-FID, and the results are summarized in Table 1. The results enabled us to identify and compare the constituent fatty acids of total lipids extracted from seeds of the *O. ficus indica *(Table 1). The percentage of each compound was calculated from the peak areas given by the GC- FID. The SC-CO_2_ extraction of fatty acids profile showed eleven peaks (Figure 3(a)); the soxhlet *n*-hexane extract of fatty acids profile illustrated only 8 peaks (Figure 3(b)). Each peak corresponded to fatty acid which was identified with respect to standards.

The main components for two forms were linoleic acid: SC-CO_2_ (spiny 57.60%, thornless 59.98%) and soxhlet *n*-hexane (spiny 57.54%, thornless 60.66), oleic acid: SC-CO_2_ (spiny 22.31%, thornless 22.40%) and soxhlet *n*-hexane (spiny 25.28%, thornless 20.58%) and palmitic acid: SC-CO_2_ (spiny 14.3%, thornless 12.92%) and soxhlet *n*-hexane (spiny 11.33%, thornless 31.08%). According to the Ramadan and Mörsel work [7], solvent extraction using a chloroform/methanol showed that palmitic acid was the second compound after linoleic acid in terms of amount. Ennouri et al. [2] reported that the oils extracted with hexane using a soxhlet extractor, linoleic acid contents were higher than our results (70.3%), oleic acid was very much lower (16.8%) and palmitic acid (9.3%). Coşkuner and Tekin [17] reported that the seed oil extracted from Turkish *O. ficus indica* (Mersin) with petroleum ether by soxhlet extractor, palmitic acid content (12%), and linoleic (52%). The observed difference is probably due to extraction process and the maturation stage of fruits. Coşkuner and Tekin [17] suggested that there was an increase in saturated fatty acid content towards the end of fruit maturation. The solvent extraction of *O. ficus indica* seeds has been studied at different conditions previously, but, to the best of our knowledge, there is not any research about the SC-CO_2_ extraction of Tunisian *O. ficus indica*. The results of SC-CO_2_ extract showed that the saturated fatty acids composition was slightly more important for wild spiny form (14.30% for palmitic acid and 3.12% for stearic acid) than the cultivated thornless form (12.92% for palmitic acid and 2.38% for stearic acid). In contrast, the soxhlet *n*-hexane extract of palmitic acid was most important for the thornless form (13.08% for) than the spiny form (11.33%). For the extraction process, the results showed that the oils were found to be highly unsaturated (*US* = 4.44–5.25). The soxhlet *n*-hexane extract showed an US more important for the spiny form (5.22), the soxhlet *n*-hexane extract allowed a better extraction of unsaturated fatty acids for the wild form. The SC-CO_2_ extract showed an US more important for the thornless form (5.25), and the SC-CO_2_ extract allowed a better extraction of unsaturated fatty acids for the cultivated form (Table 1). Compared to Ramadan and Mörsel results [7], the SC-CO_2_ profiles fatty acids of Tunisian *O. ficus indica *seeds showed the richness of this profile with other compounds such as myristic, arachidic, eicosenoic, eicosadienoic, and behenic fatty acid. Compared to our soxhlet *n*-hexane extract results, the SC-CO_2_ profiles fatty acids showed the richness of small proportion compounds such as eicosenoic, eicosadienoic, and behenic fatty acid. The profiles of fatty acids extracted from *O. dillenii* Haw. (*Cactaceae*) by supercritical CO_2_ revealed other compounds found in low percentage such as vaccenic and margaric fatty acid [18]. These results showed that the supercritical CO_2_ extraction ensures a better extraction of small proportion compounds. The composition of the spiny Tunisian* O. ficus indica* seeds oils revealed the similar fatty acid constituents at different temperature (Table 2). The temperature of SC-CO_2_ extraction seemed has not effect on the selectivity of fatty acids. The increasing temperature from 40°C (*U*/*S* = 4.44) to 70°C (*U*/*S* = 4.14) tended to decrease the SC-CO_2_ extraction yield but not affect the solubility of fatty acids.

## 4. Conclusion

The SC-CO_2_ and soxhlet *n*-hexane extraction was applied to the extraction of oil seeds from wild and cultivated Tunisian *O. ficus indica*. The oil yields were higher by soxhlet *n*-hexane extraction, and the wild form gave the best results. The fatty acids profiles were investigated, and linoleic acid was the major compound followed by oleic acid and palmitic acid for the two forms and for the two extraction process. The SC-CO_2_ extract allowed a better extraction of unsaturated fatty acids for the cultivated form, and oils revealed a better extraction of small proportion compounds. In contrast, the soxhlet *n*-hexane extract allowed a better extraction of unsaturated fatty acids for the wild form, and the fatty acids profile did not identify some compound of lower proportion. The low extractable compounds of fatty acids and the purity of the SC-CO_2_ extract are giving a better quality of oil. We can extend our research on the various parameters of supercritical extraction for optimizing better extraction efficiency. With a better extraction of small proportion compounds of fatty acids seed oils, the SC-CO_2_ process could be more advantageous than the soxhlet *n*-hexane. The fatty acids seed oil would seem to have a potential use and be valuable in different areas of bioindustrial implementation, nutraceutical and pharmaceutical. These results provide a better opportunity to improve and to enhance the natural heritage that characterizes the Tunisian *O. ficus indica.*

## Figures and Tables

**Table 1:** Relative content and results of GC-FID analysis of seeds oil fatty acid extracted by SC-CO_2_ and soxhlet *n-*hexane of two Tunisian *O. ficus indica* forms.

Tunisian *O. ficus indica* seeds	Relative content [%] *M* ± SD			
	SC-CO_2_ extract (*T* = 40°C, *P* = 180 bar, CO_2_ flow rate = 15 mL·s^−1^ and time = 135 min)		Soxhlet *n*-hexane extract	
Fatty acids	Forms			
	Spiny	Thornless	Spiny	Thornless
Extraction yield	3, 40 ± 0, 14	1,94 ± 0,09	10,32 ± 0,09	8,91 ± 0,07
Myristic C_14 : 0_	0,19 ± 0,02	0,13 ± 0,02	0,11 ± 0,01	0,07 ± 0,01
Palmitic C_16 : 0_	14,30 ± 0,24	12,92 ± 0,45	11,33 ± 0,67	13,08 ± 0,88
Palmitoleic C_16 : 1_	0,97 ± 0,03	0,85 ± 0,06	0,66 ± 0,03	0,60 ± 0,01
Stearic C_18 : 0_	3,12 ± 0,04	2,38 ± 0,09	4,27 ± 0,26	4,09 ± 0,24
Oleic C_18 : 1_	22,31 ± 0,24	22,40 ± 0,53	25,28 ± 1,08	20,58 ± 0,42
Linoleic C_18 : 2n6_	57,60 ± 0,25	59,98 ± 0,22	57,54 ± 0,40	60,66 ± 0,73
Linolenic C_18 : 3n3_	0,23 ± 0,01	0,26 ± 0,03	0,45 ± 0,04	0,55 ± 0,01
Arachidic C_20 : 0_	0,37 ± 0,05	0,26 ± 0,03	0,36 ± 0,01	0,38 ± 0,05
Eicosenoic C_20 : 1_	0,32 ± 0,03	0,30 ± 0,02	nd	nd
Eicosadienoic C_20 : 2_	0,20 ± 0,03	0,21 ± 0,02	nd	nd
Behinic C_22 : 0_	0,39 ± 0,04	0,31 ± 0,04	nd	nd
*U/S*	**4,44**	**5,25**	**5,22**	**4,68**

*M*: mean (*n* = 3); SD: relative standard deviation; *U/S*: Unsaturation ratio, *U/S* = (C_16 : 1_ + C_18 : 1_ + C_18 : 2n6_ + C_18 : 3n3_ + C_20 : 1_ + C_20 : 2_)/(C_14 : 0_ + C_16 : 0_ + C_18 : 0_ + C_20 : 0_ + C_22 : 0_); nd: not detected.

**Table 2:** Fatty acid constituents in seed oil extracted by SC-CO_2_ from Tunisian *O. ficus indica *spiny form at different temperatures at fixed parameters (*P* = 180 bar, CO_2_ flow rate = 15 mL s^−1^, and time = 135 min).

Fatty acids	Extraction SC-CO_2_ temperature [°C]			
	40	50	60	70
	Fatty acids constituents [%] *M* ± SD			
C_14 : 0_	0.19 ± 0.02	0.21 ± 0.03	0.20 ± 0.02	0.27 ± 0.03
C_16 : 0_	14.30 ± 0.24	13.93 ± 0.44	14.28 ± 0.47	15.91 ± 0.48
C_16 : 1_	0.97 ± 0.03	0.86 ± 0.02	0.94 ± 0.05	1.07 ± 0.07
C_18 : 0_	3.12 ± 0.04	3.32 ± 0.14	3.21 ± 0.08	2.59 ± 0.11
C_18 : 1_	22.31 ± 0.24	23.03 ± 0.47	22.99 ± 0.23	21.85 ± 0.52
C_18 : 2n6_	57.60 ± 0.25	56.98 ± 0.32	57.00 ± 0.18	56.33 ± 0.83
C_18 : 3n3_	0.23 ± 0.01	0.29 ± 0.05	0.25 ± 0.04	0.37 ± 0.06
C_20 : 0_	0.37 ± 0.05	0.33 ± 0.04	0.28 ± 0.03	0.27 ± 0.08
C_20 : 1_	0.32 ± 0.03	0.41 ± 0.026	0.32 ± 0.05	0.33 ± 0.05
C_20 : 2_	0.20 ± 0.03	0.30 ± 0.04	0.21 ± 0.02	0.21 ± 0.03
C_22 : 0_	0.39 ± 0.04	0.53 ± 0.02	0.2 ± 0.01	0.3 ± 0.03
*U/S*	4.44	4.45	4.49	4.13

*M*: mean (*n* = 3); SD: relative standard deviation; *U/S*: Unsaturation ratio.

## Abbreviations

SC-CO_2_: Supercritical carbon dioxide
SFE: Supercritical fluid extraction
O: *Opuntia*
GC-FID: Gas chromatography flame ionization detector.
